# Effect of nitrogen and zinc nanofertilizer with the organic farming practices on cereal and oil seed crops

**DOI:** 10.1038/s41598-022-10843-3

**Published:** 2022-04-28

**Authors:** Anil Kumar, Kapur Singh, Pushpendra Verma, Omkar Singh, Aashish Panwar, Tarunendu Singh, Yogendra Kumar, Ramesh Raliya

**Affiliations:** 1 Krishi Vigyan Kendra, Rampura, Rewari, Haryana India; 2 IFFCO Haryana State, B S Nakai Bhawan, Sector Madhya Marg, Chandigarh, India; 3 Indian Farmers Fertiliser Cooperative Limited, New Delhi, India; 4 IFFCO-Nano Biotechnology Research Centre, Kalol, Gujarat India

**Keywords:** Plant sciences, Environmental sciences, Nanoscience and technology

## Abstract

Sustainable and precision agriculture practices are essential to meet the global food demand with minimal impact on soil, air and water. In the present study, nanofertilizers of nitrogen and zinc was used with the organic farming practice under field condition for the cereal i.e. wheat, pearl millet, and oil seed crops i.e. mustard, sesame. The field trial was compared with chemical fertilizer based agricultural settings. A total of 160 field demonstrations were conducted at two locations: Khaliyawas (28.19° N, 76.76° E) and Khatawali (28.22° N, 76.76° E) of Haryana, India with a total area of 1225 acre and randomized block design. It was found that an average yield was recorded 5.35% higher in wheat, 24.24% higher yield in sesame, 4.2% higher in pearl millet and 8.4% higher yield in mustard by applying nanofertilizers of nitrogen and zinc along with the organic farming practice. The increased yield corroborated with the development parameters of plants such as wheat tillers, ear head length of pearl millet, capsule number per plant in sesame and siliquae number per plant in mustard. The trial observation suggests that the fields with applied organic manure, bio-fertilizer and nanofertilizers in combination resulted in higher yield and better plant growth performances when compared to the fields under conventional chemical fertilizer practice. The results suggest that the intervention of nanotechnology along with organic farming practice can help in minimizing the mass volume requirement of conventional chemical fertilizer while improving crop production.

## Introduction

The use of fertilizer is being practiced to produce enough food for increasing population. However, the fertilizers, particularly nitrogen (N) and phosphate (P) being used in many fold excess due to their low use efficiency and availability in the preferred chemical form, uptake by plants^1,2^. The typical use efficiency of nitrogen fertilizer (urea) is about 30–40% and phosphate is about 15–20% in most agriculture settings^3^. The unutilized fertilizer input release to the environment and pollute soil, air, water. For instance, urea volatilize in the form of nitrous oxide, a greenhouse gas and emit in the form of ammonia contributing to the global warming and air pollution^4^. The leached urea through soil in form of nitrate affecting the drinking water quality. Moreover, use of urea affect the soil pH that further affect the uptake efficiency of essential macro and micro nutrient by the plants^5^. Similarly, unutilized phosphate get runoff and leach to water bodies wherein they contribute to eutrophication and dead zones^6,7^.

To improve the nutrient use efficiency, alternative smart agri-inputs based on the concepts of advanced chemical engineering, biotechnology, microbiology, polymer science are being developed for the control and slow release of nutrient in the soil^8–11^. However, success is limited due to varying agro-climatic conditions, plant and food demand diversity and soil nutrient profiles. World population is expected to grow over 10 billion by 2100 and Asia is the top continent by population, hence the food demand is more. Therefore, it is important to develop and adopt sustainable practices wherein adequate food can be produced while minimizing the environmental impact of less efficient fertilizers. Current efforts, such as coated fertilizers for slow release^12^, mixture or macro and micro nutrient^13^, crop diversification^14^, green manure^15^, gradual reduction of fertilizers^16^ and organic farming practices in which use of organism based fertilizer, decomposer and extract of organisms^17^ are being tested.

Since last two decades, nanotechnology is being explored to enhance the nutrient use efficiency and target delivery of nutrients to plants. Fertilizers made at nanoscale (1–100 nm) having higher surface area to volume size ratio and feature of surface functionalization along with slow or plant response based release^18,19^. For instance, zinc oxide nanofertilizers were used to mobilize native phosphorus ion soil in addition to fertilize the zinc itself^20,21^. Similarly, urea coated with hydroxyapatite was tested on rice crop with the aim to reduce the use bulk alternative fertilizer of nitrogen^22^ and apatite nanoparticles as a phosphorus fertilizer^23^. The interesting observation evidenced from the laboratory or small scale field trials of nanotechnology based fertilizer inputs was the reduction in the demand of conventional bulk alternatives while maintaining or increasing the crop productivity. This inspires the present study to investigate the influence of nanofertilizers of nitrogen and zinc along with organic farming practices wherein minimal or zero amount of chemical fertilizer is being practiced. As per the literature and authors’ knowledge, this is first of its kind report wherein nanofertilizer of nitrogen and zinc was used for crop fertilization under the large scale agronomic trials to compare chemical fertilizer versus organic farming practices with nanofertilizer. The nanofertilizers were used along with organic practice to reduce the imbalanced use of bulk fertilizer such as urea with a larger aim to demonstrate alternative practice for sustainable and precision agriculture.

The aim of the present study was to compare the tested crops (cereals i.e. wheat and pearl millet and oil crops i.e. mustard and sesame) productivity response in two practices, first conventional chemical fertilizer input and the second is organic farming practice. In the organic farming practice nanofertilizers of nitrogen and zinc was used. The goal of this study was to investigate and explore the alternative practices in which the excess use of nitrogen and phosphate input can be minimized without affecting the quality and quantity of the produce.

## Results and discussion

### Characterization of nanofertiliser: nano nitrogen and nano zinc

**Figure 1:**
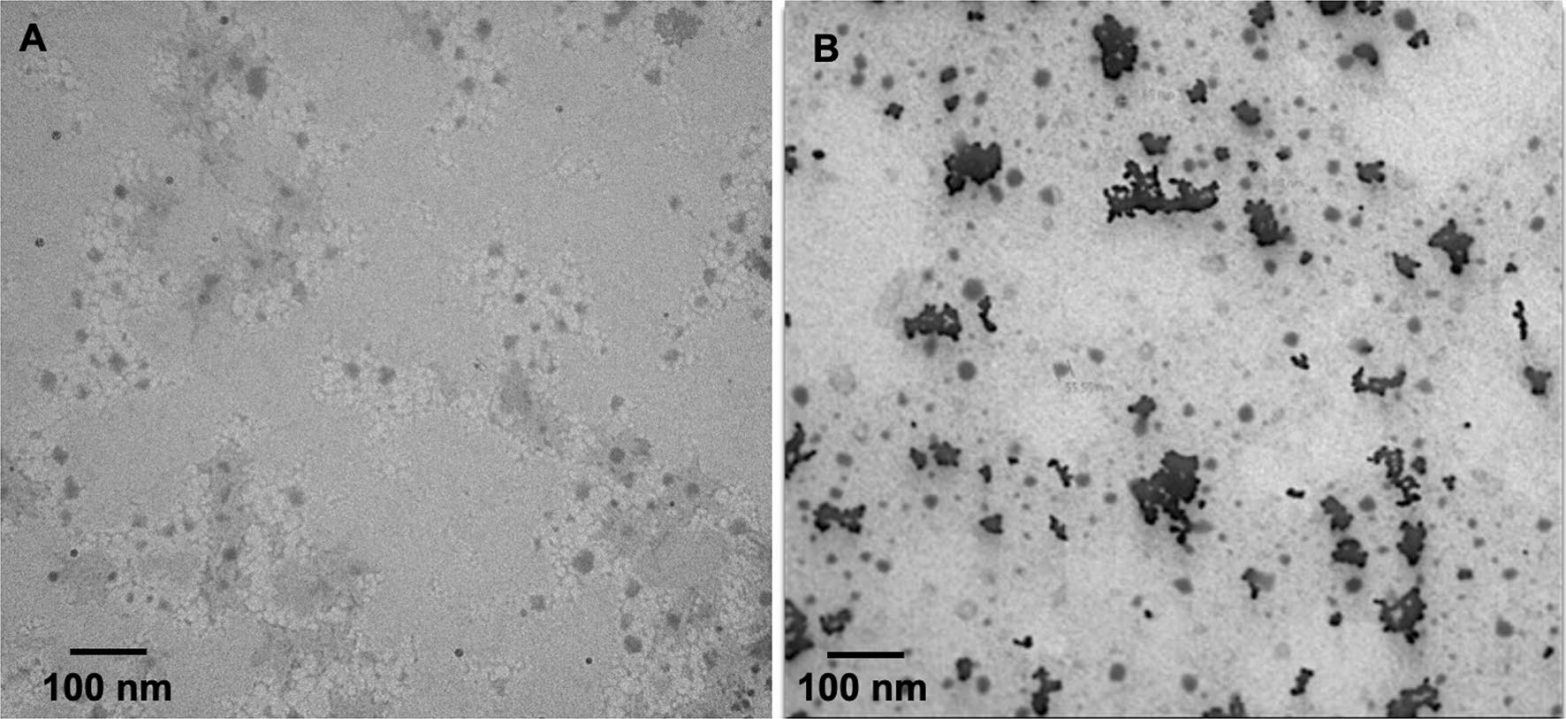
Morphological characterization of nanofertilizer: transmission electron microscope (TEM) micrograph of nano nitrogen (**A**) and nano zinc (**B**). *nm* nanometer.

Effect of nanoparticles are directly linked with its properties such as size, shape, dispersion, surface chemistry and concentration. In the present study, two type of nanoparticles were tested, one as nano nitrogen and nano zinc. The mean physical diameter of nano nitrogen and nano zinc were 28.3 and 22.3 nm, respectively (Fig. 1).

Once the nanoscale particle dispersed in liquid suspension or solvent, it is important to measure the hydrodynamic diameter and zeta potential which was 56. 6 nm and 42.4 mV, respectively for nano nitrogen and 39.4 nm and 34.2 mV, respectively for nano zinc. Further these nanoparticles were characterized for viscosity and pH which were 9.65 cPs and 4.6 respectively for nano nitrogen and 8.63 cPs and 3.2 respectively for nano zinc. Stock solution concentration of nitrogen in the nano nitrogen was 4.3% whereas total zinc in nano zinc was 1.03%. All the results, parameters and test methods are summarized in the Table 1.

**Table 1:** Characterization of nanofertilizers: nano nitrogen and nano zinc.

Parameters	Nano nitrogen	Nano zinc	Test method
Average physical size (nm)	28. 3 ± 5.8	22.3 ± 7.93	ISO 21363:2020
Average hydrodynamic size (nm)	56.6 ± 8.73	39.4 ± 12.3	ASTM E3247-20
Zeta potential (mV)	42.4 ± 1.32	34.2 ± 0.21	ISO13099-1
Viscosity (cPs)	9.65 ± 0.43	8.63 ± 0.32	ASTM D2196-10
pH	4.6 ± 0.03	3.2 ± 0.01	ASTM E70-07
Element (%)	4.3 ± 0.01	1.03 ± 0.001	ASTM D3590-02 and ASTM D8110-17

### Effect on plant growth and development

**Figure 2:**
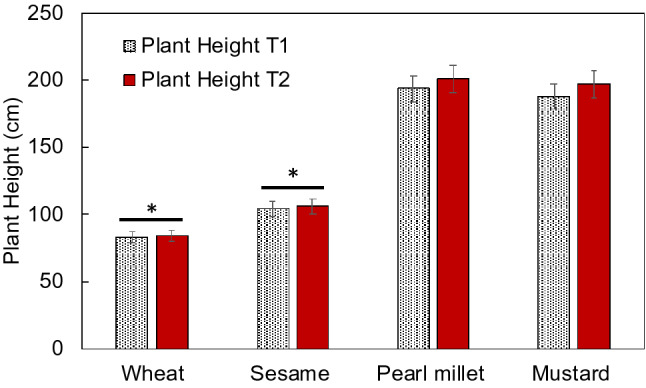
Effect on plant growth: comparison of plant height parameters of the tested crops which were grown under field condition with the treatments T1: conventional fertilizer and T: organic farming practice with nanofertilizer of nitrogen and zinc. *T1* treatment-1, *T2* treatment 2. *Non-significant difference between the treatments.

**Figure 3:**
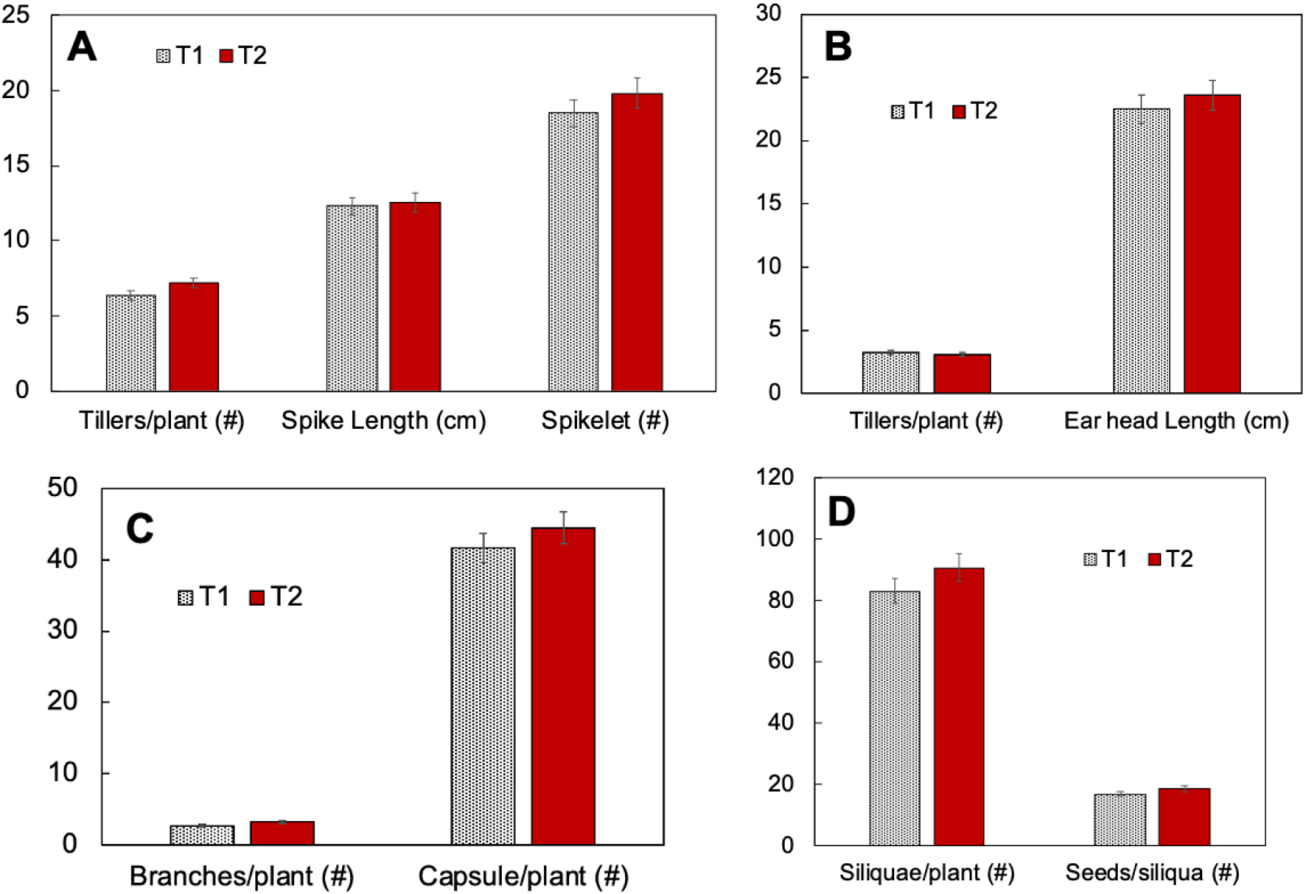
Effect on growth and development: (**A**) Wheat: tillers, spike length and spikelet (**B**) Pearl millet: tillers, ear head length (**C**) Sesame: branches, capsule (**D**) Mustard: siliquae, seeds. Parameters were observed two weeks before harvesting. *T1: conventional fertilizer and T: organic farming practice with nanofertilizer of nitrogen and zinc.* *T1* treatment-1, *T2* treatment 2.

Effect on the trial was monitored on the total of 160 field demonstrations during the year 2019–2020 and 2020–2021. Four agriculturally important crops grown in the region, wheat, pearl millet, sesame and mustard. A minimum of four replicates were maintained during the trial. To monitor the plant growth and development parameters such as height, branches or tillers are the key parameters. From the trial result, it was found that the height of wheat and sesame plants were statistically similar in the Treatment-1 (T1) and Treatment-2 (T2). However, the height was slightly more for the T2 for pearl millet and mustard crops (Fig. 2). Further, there was no significant difference in parameters such as spike length in wheat, tiller number in pearl millet, branches per plant in sesame and seeds per siliqua in mustard. However, T2 shown incremental impact on wheat for tillers per plant, 12.5% higher in T2 than T1, spikelet numbers was 7.03% higher in T2 than T1; ear head length of T2 in pearl millet was 4.9% higher than T1, capsule per plant was 6.71% higher in T2 than T1 of sesame and siliquae number per plant in mustard T2 was 9.1% higher than T1 (Fig. 3).

### Effect on grain yield

**Figure 4:**
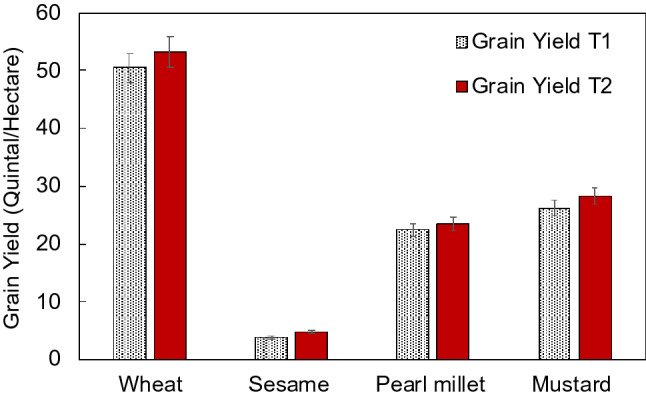
Effect on grain yield: comparison of treatments on grain yield of the tested crops which were grown under field condition with the treatments T1: conventional fertilizer and T: organic farming practice with nanofertilizer of nitrogen and zinc. *T1* treatment-1, *T2* treatment 2.

Grain yield is an ultimate economical parameter used to determine the profit or loss on the farm. It was found that an average yield was recorded 5.35% higher in T2 of wheat than T1, in sesame, 24.24% higher yield was recorded in T2 than T1. Similarly, T2 of pearl millet showed 4.2% higher yield than T1; and T2 mustard shown 8.4% higher yield than the T1 (Fig. 4). The increased yield corresponds to the development parameters such as wheat tillers, ear head length of pearl millet, capsule number per plant in sesame and siliquae number per plant in mustard (Fig. 3). One observation noted in the wheat crop during *rabi* season 2019–2020 was that after 50 days of sowing, the crop turned pale yellow in colour and showed stunted growth and less tillering but after application of nanofertilisers, the crop turned pale yellow to green coloration with vigour growth and enhanced tillering. The tested nanofertilizers kept all the crops greener for a longer period of time and as a result increased the crop maturity time which caused the crop to ripen at its proper time and promoted proper growth of the grains and the quality of grains remained high. It also prevented lodging in different crops as it strengthened the stem of the crops and as a result plants remained standing even in strong winds.

Organic cultivation adopted for various crops indicated positive effect on crop yield, economics and soil improvement. The average data revealed that soil pH decreased from 8.16 to 8.04, EC (1:2) decreased from 0.30 to 0.23 dS/m and increase in soil organic carbon was from 0.22 to 0.41 percent, available nitrogen (kg/ha) increased from 75.25 to 121.7, Available phosphorus (kg/ha) increased from 8.86 to 11.97, available potassium (kg/ha) enhanced from 147.5 to 155.4 and micronutrient i.e. zinc (mg/kg) also enhanced from 0.36 to 0.51.

**Figure 5:**
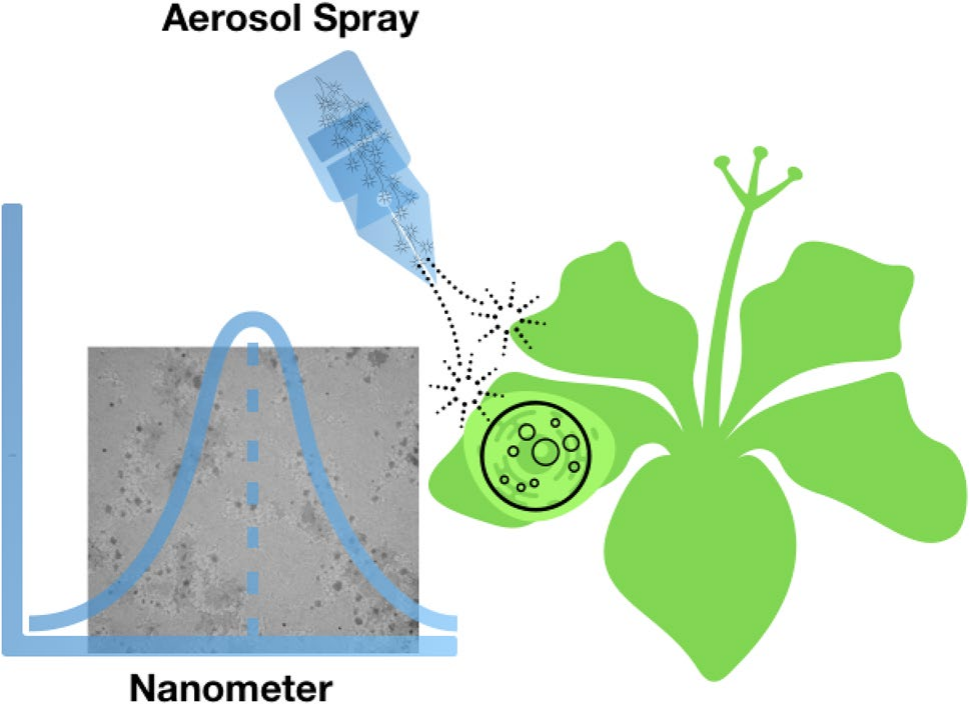
A graphical representation: nanoparticle characterization and spray on the leaves of plant.

In summary, cereals i.e. wheat and pearl millet and oil crops i.e. mustard and sesame were chosen to evaluate the productivity response in two practices, first conventional chemical fertilizer input and the second is organic farming practice. In the organic farming practice nanofertilizers of nitrogen and zinc was used by spraying on plant leaves (Fig. 5). The goal of this study was to investigate and explore the alternative practices in which the excess use of fertilizer input can be minimized without affecting the quality and quantity of the produce. A large scare on farm trial was organized for two consecutive seasons. The results of these 160 on-farm demonstrations clearly establishes the effectiveness of nutrient management concept through organic manures and nanofertilizers in the tested four crops. It was found that an average yield was recorded 5.35% higher in T2 of wheat than T1, in sesame, 24.24% higher yield was recorded in T2 than T1. Similarly, T2 of pearl millet showed 4.2% higher yield than T1; and T2 mustard shown 8.4% higher yield than the T1. The increased yield can be correlated with the development parameters such as wheat tillers, ear head length of pearl millet, capsule number per plant in sesame and siliquae number per plant in mustard. If the practices of T2 is realized in various agroclimatic conditions and translated, will have potential to increase farm income and improve environmental health by reducing the requirement of conventional or lesser efficiency conventional chemical fertilizers.

## Materials and methods

### Materials

Nanofertilizers of nitrogen and zinc nutrient element, namely Nano Nitrogen (also termed as IFFCO Nano Urea) and Nano Zinc, respectively were obtained from Nano Biotechnology Research Canter, Gandhinagar, India. The nanoparticles were dispersed in water and characterized for its size, shape, formulation pH and viscosity. Similarly, Biofertilizers, a liquid consortium of *Rhizobium, Azotobector, Azospirillum, Phosphobacteria, and potash solubilizing* bacteria were obtained from Cooperative Rural Development Trust, Kalol, India. Organic manure with a typical composition of 0.5% nitrogen, 0.2% phosphorus and 0.5% potassium were prepared at Krishi Vigyan Kendra (Agriculture Science Centre), Rampura, Haryana. Base fertilizer of N, P, K, S, Zn, Sagrika (a sea weed extract based fertilizer) were obtained from Indian Farmers Fertilizer Cooperative Limited, India. Seeds of the tested crops i.e. wheat (variety-*HD-2967*), pearl millet (*Pioneer PHI 86 M 90*), sesame (*RT 351*) and mustard (*Giriraj-DRMRIJ-31*) were obtained from local seed supplier. Further, all the experiments on the tested plants and on trial location followed the national guideline with respect to the use of seed varieties and type of agri-inputs.

### Characterization of nanofertilizers: nano nitrogen and nano zinc

The nanomaterials formulation of nano nitrogen and nano zinc were characterized for its morphology by Transmission Electron Microscopy (test method: ISO 21363:2020), hydrodynamic size (test method: ASTM E3247-20) and zeta potential (test method: ISO13099-1) by dynamic light scattering, pH by pH meter (test method: ASTM E70-07) and Viscosity by rotational viscometer (test method: ASTM D2196-10). Elemental concentration of Nano Nitrogen was obtained as Total Nitrogen Percentage by weight using the test method, ASTM D3590-02. Similarly, Zinc concentration in Nano Zinc formulation was estimated using Inductively Coupled Plasma–Mass Spectroscopy (ICP–MS) using the test method ASTM D8110-17.

### Trial locations

A total of 160 field demonstrations conducted in the Rewari district at two locations: Khaliyawas (28.19° N, 76.76° E) and Khatawali (28.22° N, 76.76° E) of Haryana, India. The trial was conducted in winter and summer seasons of the year 2019–2020 and 2020–2021. The tested crops were wheat (variety-*HD-2967*), pearl millet (*Pioneer PHI 86 M 90*), sesame (*RT 351*) and mustard (*Giriraj-DRMRIJ-31*). The total area of the test fields were 1225 acres out of which the cultivated area is 1017 acres which comes under manual irrigated. The soil types of the test locations is sandy loams soil. A minimum of four replicates were maintained in the randomized block design during the trial. In each season, the crops were sown with two different treatments, details are summarized in the Table 2.

**Table 2:** Tested crops and treatment details.

Test #	Crop	Treatment option chosen per hectare	
		Treatment 1 (T1)	Treatment 2 (T2)
1	Wheat	N:P:K:Zn (kg) (150:60:30:25)	Organic manure 2.5 MT + Biofertilizer consortium 1250 ml + Sagarika granular 25 kg + Sagarika Liq. 625 ml + Three sprays each of Nano Nitrogen and Nano Zinc
2	Pearl millet	N:P:K:Zn (kg) (60:30:0:0)	Organic manure 2.5 MT + Biofertilizer consortium 1250 ml + Sagarika granular 25 kg + Sagarika Liq. 625 ml + Three sprays of Nano Nitrogen and Nano Zinc
3	Mustard	N:P:K:Zn:S (kg) (80:30:20:25:25)	Organic manure 1.25 MT + Biofertilizer consortium 1250 ml + Sagarika granular 25 kg + Sagarika Liq. 625 ml + Three sprays each of Nano Nitrogen and Nano Zinc
4	Sesame	N:P:K:Zn (kg) (37.5:0:0:0)	Organic manure 1.25 MT + Biofertilizer consortium 1250 ml + Sagarika granular 25 kg + Sagarika Liq. 625 ml + Two sprays each of Nano Nitrogen and Nano Zinc

Base fertilizer of N, P, K, S, Zn, Sagrika were obtained from Indian Farmers Fertilizer Cooperative Limited. Nano fertilizers of N, Zn and Cu were used at the rate of 2.5 ml/l. Organic manure (i.e. farm yard manure) with a typical composition of 0.5% nitrogen, 0.2% phosphorus and 0.5% potassium.

*N* nitrogen as ammonia or nitrate, *P* phosphorus as P_2_O_5_, *K* potassium as K^+^, *Zn* zinc as zinc sulphate, *S* sulphur as SO_4_^2−^.

### Delivery of nanofertilizers: nano nitrogen and nano zinc

As received nanofertilizers were characterized and diluted with an effective concentration of nano nitrogen as 100 ppm and nano zinc as 20 ppm. The diluted nanoformualtions were mixed just before the use. The nanoformulation were foliar sprayed on the plant leaves three times in the life cycle of the plant.

### Wheat growth condition

The field trial on Wheat crop were carried out under organic cultivation. Farms were prepared as per the recommendation of the state agricultural department based on soil health survey. Organic manure at the rate 2500 kg/ha, bio-fertilizer at the rate 1.25 l/ha and Sagarika (a sea weed based bio stimulant) at the rate 25 kg/ha in granular form as soil application. Bio-decomposer was also applied at the time of pre sowing irrigation. First spray of liquid Sagarika at the rate 2.5 ml/l and Nano nitrogen and Nano zinc at the rate 2.5 ml/l was applied at 30 days after sowing, second spray was done at 45 days after sowing and the third foliar spray was applied at 60 days after sowing.

### Pearl millet and Sesame growth condition

The field trial on Pearl millet and sesame crop trials were carried out under organic cultivation. Farms were prepared as per the recommendation of the state agricultural department based on soil health survey. Organic manure at the rate 2500 kg/ha, bio-fertilizer at the rate 1.25 l/ha and Sagarika at the rate 25 kg/ha in granular form as soil application for the pearl millet crop. For sesame crop all preparation were same as wheat and pearl millet except for the rate of organic manure which was 1250 kg/ha. Bio-decomposer was also applied at the time of pre sowing irrigation in both the crops. First spray of liquid Sagarika at the rate 2.5 ml/l and Nano nitrogen and Nano zinc both at the rate 2.5 ml/l was applied at 25 days after sowing and second spray was applied at 40 days after sowing in both the crops (“Supplementary information”).

### Mustard growth condition

To take a trial on the mustard, farms were prepared as per the recommendation of the state agricultural department based on soil health survey, in which organic manure in the form of organic manure at the rate 1250 kg/ha was added along with bio-fertilizer at the rate 1.25 l/ha and sagarika at the rate of 25 kg/ha in granular form. First spray of liquid sagarika at the rate 2.5 ml/l and Nano nitrogen and Nano zinc at the rate 2.5 ml/l was applied at 30 days after sowing, second spray was done at 45 days after sowing and the third foliar spray was applied at 60 days after sowing.

### Test parameters

To study the impact of nitrogen and zinc nanofertilizer, various test parameters with respect to plant growth and development were measured. The parameters include plant height and yield of all four crops i.e. wheat, pearl millet, sesame and mustard were measured. Furthermore, crop specific parameters such as, number of tillers, spike length and spikelet were monitored for wheat; tillers and ear head length for pearl millet; branches and capsule for sesame, and sliliquae number and seeds per siliqua were observed for mustard. All the data presented are average of two years trial at the tested farm fields.

### Statistical analyses

All the sample measurements were performed in n = 4, and statistical analyses were performed using Microsoft Excel V.16.49 software. The significant differences among treatment groups were determined using the Turkey Kramer HSD at p < 0.05.

## Supplementary Information

Supplementary Information.
