# Supplementary material for: Effect of nitrogen and zinc nanofertilizer with the organic farming practices on cereal and oil seed crops
Source: Sci Rep. 2022 Apr 28;12:6938. doi: 10.1038/s41598-022-10843-3 (PMC9050747; doi:10.1038/s41598-022-10843-3)
Supplement: Supplementary file 1 — Supplementary Information. [file 41598_2022_10843_MOESM1_ESM.docx]

**Supplementary information file –**

**Effect of nitrogen and zinc nanofertilizer with the organic farming practices on cereal and oil seed crops**

**Anil Kumar^1 **^, Kapur Singh^1^, Pushpendra Verma^2^, Omkar Singh^2^, Aashish Panwar^2^, Tarunendu Singh^3^, Yogendra Kumar^3^, and Ramesh Raliya^3, 4 *^**

**^1^**Krishi Vigyan Kendra, Rampura, Rewari, Haryana, India

**^2^**IFFCO Haryana State, B S Nakai Bhawan, Sector Madhya Marg, Chandigarh, India

**^3^**Indian Farmers Fertiliser Cooperative Limited, New Delhi, India

**^4^**IFFCO-Nano Biotechnology Research Centre, Kalol, Gujarat, India

**Revised Manuscript Submitted to**

Scientific Reports

Date: March 18, 2022

**Corresponding author Email.**

* [rameshraliya@iffco.in](mailto:rameshraliya@iffco.in) (Ramesh Raliya)

** [anilyadav878@gmail.com](mailto:anilyadav878@gmail.com) (Anil Kumar)

**Index**

- **Crop wise additional observation during the trial.**
- **Soil properties and meteorology data**

**Crop wise additional observation during the trial -**

**Wheat (2019-20)**

The compiled data of wheat crop trials revealed that growth attributes *viz*. number of effective tillers per plant, plant height (cm). spike length (cm) and number of spikelet per spike were higher in T2 treatment as compared to treatment T1. Economic return of wheat increased with B:C ratio (2.75). Maximum net return obtained in T2 treatment was Rs. 102580 per hectare as compared to T1 treatment *i.e.* Rs. 51656 per hectare. One important observation noted was that 50 DAS wheat crop turned pale yellow in colour and showed stunted growth and less tillering but after application of nanofertilisers, the crop turned pale yellow to green coloration with vigour growth and enhanced tillering.

**Wheat (2020-21)**

Data emanating from 50 trials of wheat crop revealed that growth attributes *viz.* number of effective tillers per plant, plant height (cm), spike length (cm) and no. of spikelet per spike were higher in T2 treatment as compared to treatment T1. Grain yield of wheat increased by 5.35 percent with B:C ratio (3.21). Maximum net return obtained in T2 treatment was Rs. 120008 per hectare as compared to T1 treatment i.e. Rs. 56563 per hectare.

**Pearl Millet**

The compiled data of pearl millet crop revealed that growth attributes *viz*. number of effective tillers per plant,ear head length (cm) and test weight (g) were higher in T2 as compared to T1. Grain yield of pearl millet increased by 4.22 percent with B: C ratio (2.30). Maximum net return obtained in T2 treatment was Rs. 34230 per hectare as compared to T1 treatment i.e. Rs. 31825 per hect

**Sesame**

The compiled data of sesame crop revealed that growth attributes *viz.* plant height, number of branches per plant and numbers of capsules per plant were higher in T2 as compared to T1. Grain yield of sesame increased by 24.24 percent with B:C ratio (1.68). Maximum net return obtained in T2 treatment was Rs. 17120 per hectare as compared to T1 treatment i.e. Rs. 9600 per hectare.

**Mustard**

The compiled data of mustard crop revealed that growth attributes *viz*. plant height, number of siliquae per plant and number of seeds per siliqua and test weight (g) in T2 were higher as compared to T1. Yield of mustard increased by 8.40 percent with B:C ratio (3.98). Maximum net return obtained in T2 treatment was Rs. 112702 per hectare as compared to T1 treatment i.e. Rs. 85997 per hectare. Due to high oil content percentage farmers received higher selling price at the rate Rs. 500 per qt. in T2 plot as compared to T1 plot.

**Soil properties**

The experimental soil was sandy loam in texture with pH(1:2) 8.16 and EC (1:2) 0.30. The soil was low in organic carbon content (0.22%). The soil was low in available nitrogen (75.25 kg/ha), low in available phosphorus (8.86 kg/ha) and medium in potassium (147.5 kg/ha).

**Meteorology Data:**

Average Rainfall: 400-500 mm

Average Temperature: 21.14 (November to March)

30.5 (June to September)

Relative humidity: 52.8 (November to March)

60.75 (June to September)
